# Emerging of Uncommon Chronic Mastitis From *S. gallolyticus* and *S. chromogenes* in a Smallholder Dairy Farm in Cambodia

**DOI:** 10.1155/tbed/3621605

**Published:** 2025-03-03

**Authors:** Nouv Sophorn, Na Sambo, Satoshi Ohkura, Sho Nakamura, Shuichi Matsuyama, Tetsuma Murase, Rin Soriya, Witaya Suriyasathaporn

**Affiliations:** ^1^Asian Satellite Campuses Institute, Nagoya University, Nagoya, Japan; ^2^Department of Animal Health and Veterinary Public Health, General Directorate of Animal Health and Production, Phnom Penh, Cambodia; ^3^Faculty of Veterinary Medicine, Chiang Mai University, Chiang Mai, Thailand; ^4^Laboratory of Animal Production Science, Graduate School of Bioagricultural Sciences, Nagoya University, Nagoya, Japan; ^5^Laboratory of Veterinary Theriogenology, Faculty of Applied Biological Sciences, Gifu University, Gifu, Japan

**Keywords:** chronic mastitis, mastitis, mixed infection, *S. chromogenes*, *S. gallolyticus*

## Abstract

The complete mastitis control program is insufficient for the starting dairy industry country, and therefore it might cause emerging of new mastitis pathogens. This longitudinal study aimed to determine the association of the infected dynamic status of the main pathogens responsible for mastitis with seasonal variations, the proportions of transient and chronic intramammary infection (IMI) episodes, and the duration of IMI. This study was conducted on a training smallholder dairy farm in Phnom Penh, Cambodia, from January 2023 to July 2023. Trained veterinarians aseptically collected quarter milk samples from all milking cows (*n* = 21) every 2 weeks until the end of the study, accounting for 3–16 times of milk collection per cow based on their period of lactation. All collected milk samples (*n* = 812) were cultured, and subsequently, all bacterial colonies were identified using a MALDI-TOF mass spectrometer. An IMI episode is defined as a sequence of consecutive isolates of a specific bacterium from the same quarter. The duration of an episode is the time between the new IMI and its cure. Two types of IMI were defined as transient IMI and chronic IMI that lasted for 28 days or more. Results of the IMI episodes, distributions of no, single, double-mixed, and 3-mixed IMI were 61.1%, 31.9%, 6.3%, and 0.7%, respectively, in which the mixed IMI accounts for 18% of IMI samples. *Streptococcus uberis*, *Staphylococcus chromogenes*, and *Streptococcus gallolyticus* were the main organisms responsible for the mastitis epidemic on this farm. These bacteria had higher ratios of chronic episodes than the other mastitis bacteria found on this farm. In addition, results obtained from Cox's model showed that *S. chromogenes* had a longer time to cure than pathogens other than *S. uberis* and *S. gallolyticus*, in which *S. gallolyticus* linked to colon neoplasia in humans. In conclusion, the lack of an optimal mastitis control program, in this case, provides information on the emerging mixed infections, emerging mastitis pathogens, and emerging chronic *S. chromogenes* infections.

## 1. Introduction

Milk, a rich source of essential nutrients, is a crucial component of a healthy diet. Recognizing its importance, international healthcare programs advocate for milk consumption. In 2021, Cambodia, the 30th largest milk importer in the world, imported milk worth $ 60.4 million [1], leading to high milk prices in the country. To address this, the Cambodian government has taken proactive steps by promoting local dairy production as part of its strategy [1]. This initiative is not just about reducing milk prices but also about ensuring food security and promoting economic growth. As a result, some dairy farms have begun supplying locally produced milk to major supermarkets in Phnom Penh in 2022. However, the country's healthcare knowledge and services related to dairy cattle still need to be improved, posing a significant challenge to the sustainability of this initiative.

Mastitis, a disease that inflicts significant economic damage on the dairy industry globally, is a pressing concern. The most prevalent cause of mastitis is the intramammary infection (IMI) of mastitis bacterial pathogens. These pathogens can either be contagious, spreading from cow to cow via mammary glands, or environmental, found in the herd's surroundings such as bedding materials, manure, and soil. While mastitis can be self-cured or spontaneously cured using the udder defense mechanism [2], the contagious mastitis pathogens have less spontaneous cure rates and a higher probability of chronic or persistent IMI [3, 4]. The implementation of a standard mastitis control program is therefore not just crucial but imperative to prevent and control mastitis. This program should require proper prevention of IMI and effective control methods, such as good milking procedures, teat-dipping antiseptics, and proper treatment for chronic mastitis with antibiotics [5–7]. The potential benefits of this program for dairy farms are immense, as it can significantly reduce the incidence of mastitis and its long-term economic impact.

Various studies have indicated that the most recurrently occurring pathogens in cases of mastitis worldwide are *S. aureus*, *S. agalactiae*, *S. uberis*, *E. coli*, and *K. pneumoniae* [8–10]. Poorly implemented mastitis control programs tend to encourage infections from a variety of bacteria, resulting in mixed infections [11]. Mixed infections of two or more mastitis pathogens were also detected more commonly by polymerase chain reaction (PCR) tests [12]. Bacteria present in the mammary glands as the areas of the host organism's environment utilize competitive mechanisms to survive and shape the composition and function of the diverse community [13]. Dominant mastitis pathogens are typically seen in studies on bacterial competition [3, 14]. However, there needs to be more information concerning mixed IMIs in herds with insufficient mastitis control programs. Additionally, IMI bacteria pathogens in the new dairy industry environment or mixed IMIs might differ from those found in other parts of the world with different farm management systems.

In the last decades, MALDI-TOF MS has emerged as a fast microbial identification approach [15] and is being successfully applied to most bacteria isolated from bovine milk [15–18]. In addition, MALDI-TOF is valuable for the identification and typeability of non-Aureus Staphylococci (NAS) [18, 19]. The present longitudinal study was conducted to delineate the dynamics of IMI, focusing particularly on mixed IMI prevalent in a smallholder dairy farm situated in Cambodia using MALDI-TOF assay for bacterial identification. The study aimed to determine the association of the infected dynamic status of the main pathogens responsible for mastitis, that is, uninfected, newly infected, and persistently infected IMI, with seasonal variations. In addition, the study compared the proportions of transient and chronic IMI episodes and the duration of IMI between the main mastitis pathogens. This investigation is valuable in enhancing our understanding of the dynamics of IMI, thus facilitating the development of effective interventions for preventing and controlling mastitis in dairy cattle.

## 2. Materials and Methods

### 2.1. Farms, Animals, and Study Design

This study was conducted in Phnom Penh, Cambodia, on a train-smallholder dairy farm in a public university, using all milking cows from January 2023 to July 2023. The average milk yield ranged between 9.5 and 11 kg/cow/day. Three veterinarians were responsible for managing and preventing diseases, but the mastitis control program was initiated after the study began. The cows were crossbred Holstein-Friesian and were housed in a free-stall facility with concrete floors. They were milked twice a day using a 16-milking unit pipeline instrument in the separated milking parlor. The cow udder was washed with water and dried with a separate clean towel before milking. Due to the limited availability of dairy cow products, a local iodine disinfectant was used as a postdipping antiseptic agent, and no California Mastitis Test (CMT) was used to monitor subclinical mastitis in the herd. Acute and per-acute clinical mastitis were treated with systemic administration of antibiotics because there was a lack of intramammary antibiotic infusion in the market. There was no dry-cow therapy device available in the country. All of the milk from the farm was the sole source of raw milk for a self-pasteurizing milk company.

**Figure 1:**
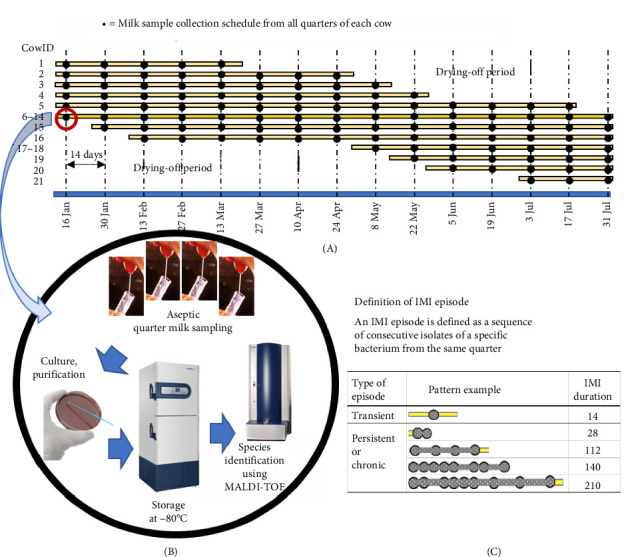
The study diagram is separated into three parts: (A) a schedule of sample collection, (B) laboratory procedures, and (C) interpretation of the IMI episode. IMI, intramammary infection.

The study included all 21 milking cows. Trained veterinarians aseptically collected quarter milk samples every 2 weeks until the end of the study, as shown in Figure 1. The samples were transported with ice to the Laboratory of Bioagricultural Sciences Nagoya University-Cambodia Satellite Campus within 2 h for bacterial culture. Milk samples were cultured on a 5% blood agar plate at 37°C for up to 24 h. An IMI was identified when a single sample contained ≥1000 colony-forming units (cfu)/ml of one or two pathogens or when a sample of a clinical case contained ≥100 cfu/ml [3, 20]. Samples with more than three different colony morphology bacteria were considered contaminated and were not informative of IMI status. Each different morphology bacterial colony was subcultured in a 5% blood agar plate at 37°C for up to 24 h until its purification. The bacterial colonies were stored at −80°C until use.

### 2.2. MALDI-TOF MS for Bacterial Identification

The bacterial isolates were obtained from frozen archives and placed in tryptone soy broth (TSB). They were then incubated overnight at 37°C. After that, the samples were subcultured and purified on tryptone soy agar (TSA) and incubated at 37°C for 24 h. The bacterial colonies were submitted to the Office of Scientific Instrument and Testing at Prince of Songkhla University, Songkhla, Thailand, for bacterial identification using a MALDI-TOF mass spectrometer through the direct transfer protocol. The manufacturer's recommendation was followed to identify bacterial fingerprints based on intact ribosomal proteins. The spectra were analyzed using Bruker Biotype software and Real-Time Classification 3.0 software, as described [21]. Only the isolates with a species-level identification score ≥2.00 and a log score ≥2.00 based on the manufacturer's criteria were used.

### 2.3. Statistical Analysis

In a study by Leelahaponsathon et al. [4], an IMI episode is defined as a sequence of consecutive isolates of a specific bacterium from the same quarter. The duration of an episode is the time between the new IMI and the spontaneous cure. The spontaneous cure of an IMI episode is determined when an infected quarter does not show the same bacteria for at least 1 month or three immediate subsequent samplings, based on a 2-week sampling interval. The start and end dates of IMI were estimated halfway or 1 week between two adjacent sampling dates. The duration of infection was defined as the time between the midpoint between the last negative and the first positive sample and the midpoint between the last positive and the first negative sample, or the interval between the first and last IMI plus 14. The first positive sample was identified as the new infection time (New), and the subsequent samples until the last positive sample were identified as the persistent infection times (Persistent). There are two types of IMI: transient IMI and chronic IMI. Chronic IMI lasts for 28 days or more, according to the definition of chronic mastitis by Bonestroo et al. [22]. For statistical analysis, the main bacterial species must account for at least 10% of all isolates. Any other species were grouped as “Other” for analysis. Seasons were defined by sampling dates, including Cool (Jan–Feb), Hot (Mar–May), and Rainy (Jun–July).

The study presented the distribution of mastitis among different types of bacteria in terms of percentages. The proportions of uninfected, New, or Persistent infections were compared between seasons for each type of bacteria using Fisher's Exact *Chi*-square tests. Fisher's Exact *Chi*-square test was used to compare the proportion of transient and chronic episodes among bacterial groups. The duration of IMI among different types of bacteria was analyzed through survival analysis. The failed case was defined as the quarter that experienced spontaneous cure, while the censored case was defined as the quarter that was infected at the last observation. A logistic regression model was used to analyze the differences in pathogen ratios between transient and chronic episodes. The Kaplan–Meier survival function was used to describe the potential differences among different types of bacteria. The Cox proportional hazard model was used to compute the hazard ratio (HR) to compare the ratio of the spontaneous cure of the specified bacteria with the reference bacteria. The significance level was set at *p*  < 0.05, which indicates that the observed results were not likely due to chance. Additionally, a trend was observed with a *p*-value of less than 0.10, indicating a possible association between the variables that require further investigation to confirm any significant findings.

## 3. Results

**Figure 2:**
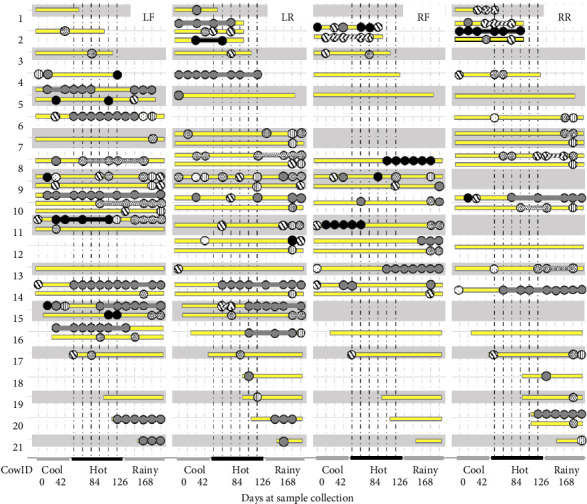
Quarter intramammary infection from aseptic milk sample collected every two weeks (vertical lines) of a longitudinal study using all milking cows in a smallholder dairy farm, Cambodia. LF Left-front quarter, LR left-rear quarter, RF right-front quarter, and RR right-rear quarter. 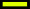 Sampling period without IMI; circles indicated the positive bacterial identified samples, including 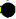
*S. gallolyticus*, 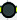
*S. chromogenes*, 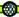
*S. uberis*, and Other pathogens, including 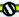 Other minor pathogens such as NAS and *Corynebacterium* spp., 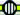 Gram-negative bacteria, and 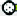 other Gram-positive bacteria; 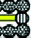,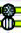 indicated 3-mixed and double-mixed IMI, respectively; a single spot indicates transient IMI and the connected spots indicates chronic IMI, and seasons, including Cool (Jan–Feb), Hot (Mar–May), and Rainy (Jun–July). The connecting line of the same bacteria indicates the chronic IMI. The end of the IMI episode is defined as when the same bacteria has not been found for three consecutive times. IMI, intramammary infection; NAS, non-Aureus Staphylococci.

From a total of 21 cows, three cows with two blinded quarters and six cows with one blinded quarter were included, and therefore, the observed quarter was 72. Cow IDs 6–14 had completed 16 collected samples 16 times at 2-week intervals, while 5 and 7 cows were incompletely collected due to drying before the end of the study or drying period at the start of the study (Figure 2). The total collected sample was 812, with an average of 53.13 samples/time ranging between 47 and 57 on days 56 and 126, respectively. Results from bacterial cultures showed that most quarters (83.3%) had at least 1 IMI, indicated by the circles in Figure 2. The LF quarter of CowID 10 at week 16 had three positive bacteria because one out of two stored bacteria of this quarter had two different colonies during the subculture. Based on the positive samples confirmed with MALDI-TOF MS, distributions of no, single, double-mixed, and 3-mixed IMI were 522 (64.3%), 251 (30.9%), 38 (4.7%), and 1 (0.1%), respectively. Results of the editing IMI episodes, as seen at the connecting line of the same bacteria (Figure 2), distributions of no, single, double-mixed, and 3-mixed IMI were 496 (61.1%), 259 (31.9%), 51 (6.3%), and 6 (0.7%), respectively, in which the mixed IMI accounts for 18% of IMI samples. All six 3-mixed IMI included at least one chronic IMI episode, as shown in CowID 2-RF Day 42 and 56, 2-RR Day 42 and 84, and CowID 10 LF Day 140 and 196. For 51 double-mixed IMI, 39 (76.5%) included at least one chronic IMI episode.

### 3.1. Bacteria Caused Mastitis and Proportions of Chronic Episodes

*S. uberis*, *S. chromogenes*, and *S. gallolyticus* were the main organisms responsible for mastitis on this farm. Out of a total of 174 episodes, the pathogens that had fewer than 17 episodes were grouped into a category called “Other.” This included other minor pathogens (*n* = 40), Gram-negative bacteria (*n* = 24), and other Gram-positive bacteria (*n* = 8). The minor pathogens included *S. haemolyticus* (13), *S. saprophyticus* (*n* = 6), *S. borealis* (*n* = 5), *S. epidermidis* (*n* = 4), *S. xylosus* (*n* = 2), *C. amycolatum* (*n* = 2), *C. falseness* (*n* = 2), and each of *S. Barletta*, *S. cohnii*, *S. hominis*, *S. simulans*, *S. warneri*, and *S. xerosis*. The Gram-negative bacteria included *E. coli* (*n* = 9), *A. baumannii* (*n* = 4), *S. marcescens* (*n* = 4), *E. bugandensis* (*n* = 2), *A. towneri* (*n* = 3), and each *E. asburiae* and *B. diminuta*, while the other Gram-positive bacteria were *S. dysgalactiae* (*n* = 2) and each of *M. caseolyticus*, *W. cibaria*, *E. faecium*, *P. muralis*, *B. pumilus*, and *P. okeanokoites*. Most pathogens in the “Other” category were transient except for 3, 1, and 1 chronic episodes of *S. haemolyticus*, *C. amycolatum*, and *C. falsenii*, respectively. The numbers of overall episodes (transient, chronic episodes) were 16 (9, 7), 40 (18, 22), 44 (28, 16), and 74 (69, 5) for *S. gallolyticus*, *S. chromogenes*, *S. uberis*, and Other, respectively. There were significant differences in the ratios of transient and chronic episodes among various pathogens (*p*  < 0.05). *S. gallolyticus* (odd ratio [OR] = 10.7), *S. chromogenes* (OR = 16.9), and *S. uberis* (OR = 7.9) had higher probabilities of chronic episodes than the pathogens in the “Other” category (*p*  < 0.05).

### 3.2. Effects of Seasons on Persistent and Transient IMI

**Figure 3:**
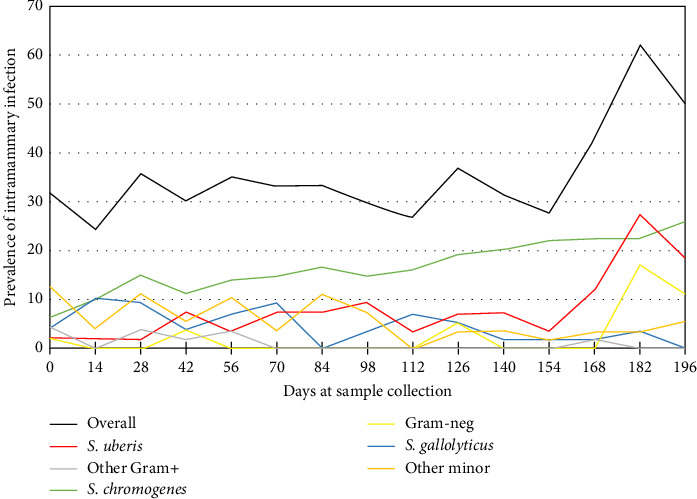
Percentages of intramammary infection among 14-day sampling period. The first sample was in January, and the last sample was in July 2023.

The percentages of IMI isolates for each sampling period are visible in Figure 3. The highest overall IMI percentage was observed during the rainy season on days 182 and 196. This was due to the highest *S. uberis* IMI and Gram-negative IMI during this period.  Table 1 shows the comparison of the New or Persistent IMI percentage of each pathogen among seasons. The New IMI of *S. gallolyticus* decreased among seasons (*p*  < 0.01), while the rest of the pathogens did not show significant association with seasons. The percentages of persistent isolates of *S. chromogenes* (22.1%) and *S. uberis* (9.7%) were significantly higher during the rainy season, indicating a significant association between IMI and season. On the other hand, the association between persistent IMI and seasons of *S. gallolyticus* was on the edge of being statistically significant (*p*=0.06).

### 3.3. Mastitis Bacteria and Duration of IMI

**Figure 4:**
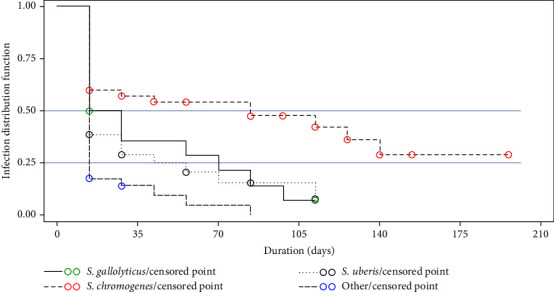
Kaplan‒Meier survival curve of intramammary infection distribution function.

Figure 4 displays a Kaplan–Meier curve that illustrates the distribution of IMI among different pathogens. The median time for a 50% spontaneous cure of IMI was found to be 14 days for *S. uberis* and Others, 21 days for *S. gallolyticus*, and 84 days for *S. chromogenes*. According to the results obtained from Cox's model, pathogens were found to be correlated with spontaneous cures. In comparison to Others, *S. chromogenes* had a significantly lower rate of spontaneous cure with HR = 0.35, while no significant differences were observed for *S. gallolyticus* and *S. uberis*.

## 4. Discussion

This study found a high prevalence of mastitis in dairy cows, ranging from 24.5% in January to 62.1% in July (Figure 3). The studied farm, located in Phnom Penh, Cambodia, needed more support in terms of knowledge and available healthcare products for dairy cattle, which might have contributed to a poor mastitis control program. The optimal procedure for controlling mastitis is a mastitis control program that takes into consideration several risk factors known to be associated with bovine mastitis [8]. General measures to prevent new cases of mastitis include improving sanitation, such as enhancing milking hygiene, implementing post-milking teat disinfection, and maintaining milking machines [23].

### 4.1. *S. gallolyticus*, an Emerging Mastitis Pathogen

In this study, 30 species of bacteria were found to be the causes of IMI. Still, only three species, *S. gallolyticus*, *S. uberis*, and *S. chromogenes*, were defined as the major cause in this herd. This study was the first report on finding *S. gallolyticus* and *S. chromogenes* as the main mastitis pathogens in a herd. For streptococci, the most relevant streptococcus species inducing bovine mastitis included *S. agalactiae*, *S. dysgalactiae*, and *S. uberis* [24]. *Streptococcus gallolyticus* belongs to the Group D streptococci, a large group of phenotypically diverse bacteria known as the *S. bovis/S. equinus* complex (SBSEC), which consist of safe-graded bacteria used in food-fermentation, commensal bacteria of the gut and opportunistic pathogens in both humans and animals [25]. *S. gallolyticus* infection in humans became a serious problem when several clinical studies demonstrated a strong association between invasive infections with *S. gallolyticus* and colon neoplasia in humans [25, 26]. *S. gallolyticus*, formerly known as *S. bovis* biotype I, is also part of the rumen biota but can cause disease in ruminants as well as in avian [27]. However, most reports indicated that *S. gallolyticus* was a rare mastitis pathogen. An isolate of *S. gallolyticus* has been reported as a mastitis pathogen in the milk of providers in Egypt [28] or one case of gangrenous mastitis caused by *S. gallolyticus* in Japan [29]. This study is the first report of a high prevalence of *S. gallolyticus* mastitis, which might be due to the poor mastitis control program on this farm in Cambodia, which is the starting dairy industry in the country. However, the use of conventional bacterial identification for mastitis [30, 31] might need to be improved in terms of the power of identifying bacterial species, especially in rare cases.

### 4.2. Emerging of the Mixed IMI

Our study revealed the dynamics of IMI, particularly a high number of mixed IMIs. Interestingly, most mixed IMIs included at least one chronic episode in those mixed IMIs. A poor mastitis control program can cause changes in regular teat scoring, resulting in increased teat-end hyperkeratosis and a higher risk of mastitis [32]. Cows with very rough teat end rings and dirty udders are more susceptible to IMI [33]. Our study also found that persistent IMI due to poor mastitis control programs could cause high mixed IMI. An in vitro study demonstrated that coculturing two mastitis pathogens resulted in one bacterium dominating the infection over time [14]. However, our study might have underestimated the prevalence of the mixed IMI in farms with high mastitis rates or poor control programs due to the exclusion of samples with three different bacteria, which were considered contaminated during the bacterial culture.

### 4.3. *S. gallolyticus* and *S. chromogenes*, Emerging Chronic Mastitis Pathogen

We found that certain groups of bacteria, including Gram-negative, NAS, and Corynebacterium, as well as other Gram-positive bacteria, had a lower risk of chronic IMI episodes compared to *S. gallolyticus* (OR = 10.7), *S. chromogenes* (OR = 16.9), and *S. uberis* (OR = 7.9). We also found that *S. haemolyticus*, a species in NAS, showed persistent episodes of IMI. Our study showed that *S. gallolyticus* had chronic IMI episodes, which is the first report of its kind (Figure 2). In humans, chronic colonization by *S. gallolyticus* is strongly associated with the occurrence of colorectal cancer, which may promote colonization of this bacteria in the gut by replacing commensal enterococci in their niche [34]. Therefore, the chronic episode of *S. gallolyticus* IMI might be due to changes in the mammary gland niche of this farm.

Our findings of chronic *S. chromogenes* IMI are supported by a previous longitudinal study in the Netherlands, which showed that 45% of IMI caused by *S. chromogenes* persisted for at least 28 days [35]. Bexiga et al. [36] found no significant differences in the mean duration of IMI caused by different NAS species. The different findings might be due to the differences in the phenotype of *S. chromogenes* strains on their survival within an environment with the other mastitis pathogens [14]. In addition, some *S. chromogenes* strains in this study appeared to be best adapted to survive in the udder gland. Persistent *S. uberis* has been reported in several studies, with some lasting more than 300 days of IMI [3, 4, 37]. Virulent *S. uberis* strains and low performance of the udder defense mechanism were suggested to be the cause of persistent *S. uberis* [4].

Table 1 reports that there were different types of IMI isolates in Cambodia during different seasons across all bacterial groups. During the study, the increased use of mastitis control programs, such as antiseptic teat dipping, dry cow therapy, and intramammary infusion, as well as subclinical mastitis monitoring with the CMT, suggested by an experienced veterinarian from Thailand, might have changed the environment of the mammary gland niche. This change might be the reason for the subsequent decrease of *S. gallolyticus*, while *S. chromogenes* and *S. uberis* became the main sources of mastitis pathogens on the farm. In general, the prevalence of mastitis varied at the international, national, and farm levels due to seasonal variations, farm management, and cow-related factors. Previous studies reported a seasonal variation in mastitis cases among different farms with high prevalence during the wet season (fall/winter) in comparison to the dry season (spring/summer) [38–40]. Other reports from tropical areas have also shown that mastitis from environmental pathogens was highest during the rainy season [41, 42].

## 5. Conclusion

A country initiating the dairy industry had limitations on knowledge and facilities for dairy health care, especially the mastitis control program. This study concluded that the limitation of the mastitis control programs caused increases in emerging uncommon IMI from the mixed infection, uncommon IMI pathogens such as from *S. gallolyticus*, and uncommon characteristics of bacteria such as the chronic IMI found for *S. chromogenes*, the NAS. The mixed infection in both double-mixed and 3-mixed IMI was obviously found in this study. Our finding warns that the new dairy industry or the insufficient performance of the mastitis control program might cause emerging uncommon mastitis problems. In addition, the inappropriate use of antibiotics might cause an antibiotic-resistant problem, which needs further study on these bacteria. To control persistent subclinical mastitis, farmers need to be strict in mastitis control programs, for example, monitoring bacteria causing mastitis, ordering milking cows, and treating with antibiotics when necessary.

## Figures and Tables

**Table 1:** Numbers (percentages) of isolates of the specified pathogens.

Pathogens	IMI series	Cool	Hot	Rainy	*p*-Value
*S. gallolyticus*	Uninfected	186	256	327	
	New	9 (4.6)	5 (1.9)	2 (0.6)	*p* < 0.01
	Persistent	7 (3.6)	14 (5.2)	6 (1.8)	*p*=0.06

*S. chromogenes*	Uninfected	176	225	243	
	New	20 (10.2)	18 (7.4)	23 (8.7)	*p*=0.58
	Persistent	6 (3.3)	32 (12.5)	69 (22.1)	*p* < 0.01

*S. uberis*	Uninfected	195	254	281	
	New	7 (3.5)	13 (4.9)	24 (7.9)	*p*=0.09
	Persistent	0	8 (3.05)	30 (9.7)	*p* < 0.01

Other	Uninfected	179	253	302	
	New	21 (10.5)	15 (5.6)	31 (9.3)	*p*=0.10
	Persistent	2 (1.1)	7 (2.7)	2 (0.7)	*p*=0.15

*Note:* Regarding the episode defined in Figure 2, New is the first positive, and Persistent is the following sample until the last positive sample of the episodes. Percentages of New or Persistent are the ratio against Uninfected, respectively.

Abbreviation: IMI, intramammary infection.

## Data Availability

The data that support the findings of this study are available from the corresponding author upon reasonable request.
